# Robust prediction of individual personality from brain functional connectome

**DOI:** 10.1093/scan/nsaa044

**Published:** 2020-04-04

**Authors:** Huanhuan Cai, Jiajia Zhu, Yongqiang Yu

**Affiliations:** Department of Radiology, The First Affiliated Hospital of Anhui Medical University, Hefei 230022, China

**Keywords:** personality, five-factor model, resting-state fMRI, functional connectome, predictive models

## Abstract

Neuroimaging studies have linked inter-individual variability in the brain to individualized personality traits. However, only one or several aspects of personality have been effectively predicted based on brain imaging features. The objective of this study was to construct a reliable prediction model of personality in a large sample by using connectome-based predictive modeling (CPM), a recently developed machine learning approach. High-quality resting-state functional magnetic resonance imaging data of 810 healthy young participants from the Human Connectome Project dataset were used to construct large-scale brain networks. Personality traits of the five-factor model (FFM) were assessed by the NEO Five Factor Inventory. We found that CPM successfully and reliably predicted all the FFM personality factors (agreeableness, openness, conscientiousness and neuroticism) other than extraversion in novel individuals. At the neural level, we found that the personality-associated functional networks mainly included brain regions within default mode, frontoparietal executive control, visual and cerebellar systems. Although different feature selection thresholds and parcellation strategies did not significantly influence the prediction results, some findings lost significance after controlling for confounds including age, gender, intelligence and head motion. Our finding of robust personality prediction from an individual’s unique functional connectome may help advance the translation of ‘brain connectivity fingerprinting’ into real-world personality psychological settings.

## Introduction

Personality is a distinctive, relatively stable and high-level psychological concept that defines individual human beings. It strongly influences long-term behavioral styles, such as social interactions, emotional expression and academic or job performance (Back *et al.,* 2009; Poropat, 2009). The five-factor model (FFM, aka ‘The Big Five’) has emerged as the leading psychometric model in the field of personality psychology (McCrae and John, 1992). A growing body of research has provided evidence that five personality factors (i.e. neuroticism, extraversion, openness, agreeableness and conscientiousness) of the FFM can well capture key descriptors of different behavioral tendencies (Heine and Buchtel, 2009). Thus, elucidating the biological basis of these personality factors holds value in gaining more insight into vulnerability and resilience, aptness for skills and areas of expertise and even facilitating a deeper understanding of our individuality as human beings.

The unbiased assessment of brain structure and function with advanced neuroimaging techniques and novel analysis approaches has linked inter-individual variability in the brain to individualized personality traits, which provides new insight into the neural correlates of personality. For example, the associations between brain structure and personality have been extensively explored by using structural magnetic resonance imaging (MRI) to measure gray matter morphology (Omura *et al.,* 2005; Rauch *et al.,* 2005; Wright *et al.,* 2006, 2007; Blankstein *et al.,* 2009; DeYoung *et al.,* 2010; Cremers *et al.,* 2011; Hu *et al.,* 2011; Schutter *et al.,* 2012; Bjornebekk *et al.,* 2013; Coutinho *et al.,* 2013; Kapogiannis *et al.,* 2013; Koelsch *et al.,* 2013; Liu *et al.,* 2013; Taki *et al.,* 2013; Forbes *et al.,* 2014; Lu *et al.,* 2014; Nostro *et al.,* 2017; Schultz *et al.,* 2017; Riccelli *et al.,* 2017b; Ferschmann *et al.,* 2018) and using diffusion MRI to evaluate white matter integrity (Xu and Potenza, 2012; Picerni *et al.,* 2013; Nenadic *et al.,* 2015; Lewis *et al.,* 2016). There are also a large number of studies investigating the associations between brain function and personality by using functional MRI (fMRI) to measure task-induced brain activation (Canli and Amin, 2002; Eisenberger *et al.,* 2005; Haas *et al.,* 2006; Gioia *et al.,* 2009; Cremers *et al.,* 2010; Suslow *et al.,* 2010; Kennis *et al.,* 2013; Koelsch *et al.,* 2013; Dima *et al.,* 2015; Riccelli *et al.,* 2017a), resting-state regional neural activity (Kunisato *et al.,* 2011; Wei *et al.,* 2011, 2014; Gentili *et al.,* 2017), functional connectivity (Adelstein *et al.,* 2011; Ryan *et al.,* 2011; Lei *et al.,* 2013; Aghajani *et al.,* 2014; Sampaio *et al.,* 2014; Kruschwitz *et al.,* 2015; Pang *et al.,* 2016; Gentili *et al.,* 2017; Tian *et al.,* 2018) and functional network topology (Gao *et al.,* 2013; Koelsch *et al.,* 2013; Lei *et al.,* 2015; Beaty *et al.,* 2016). However, these previous studies have yielded inconsistent findings with the exception of the prefrontal cortex and limbic regions (especially the amygdala and cingulate cortex), which cannot offer a compelling demonstration of the relationship between brain and personality. Moreover, most of these MRI studies have focused on establishing correlational relationships between personality traits and individual brain regions or small-scale neural circuits. However, unlike some aspects of cognitive and emotional processes that have been more or less localized to specific brain regions or circuits, personality is usually thought to be the result of a connectivity-based interaction that engages network-mediated integration across the entire brain.

The brain functional connectivity profiles have been shown to act as unique ‘neural fingerprints’ with highly individualized patterns, which allow identification of individuals at the single-subject level (Finn *et al.,* 2015; Xu *et al.,* 2016). Recently, functional connectivity patterns of the whole-brain, large-scale functional connectome have been utilized to predict individual personality traits by means of machine learning methods (Dubois *et al.,* 2018; Feng *et al.,* 2018; Hsu *et al.,* 2018). However, only one or several aspects of personality have been predicted in these studies.

In this study, we aimed to predict personality factors of the FFM at the individual level by applying a connectome-based predictive modeling (CPM) approach to high-quality resting-state fMRI data from a large sample of healthy young adults. CPM is a recently developed machine learning method for generating brain–behavior models from whole-brain functional connectivity patterns (Shen *et al.,* 2017), which has been demonstrated to reliably predict fluid intelligence (Finn *et al.,* 2015), attention (Rosenberg *et al.,* 2016; Yoo *et al.,* 2018) and creativity (Beaty *et al.,* 2018). CPM is not only a predictive tool but also a data-driven method for identifying functional networks that underlie specific behaviors. Therefore, we expected that our prediction models would effectively and reliably predict most personality factors of the FFM.

## Materials and methods

### Participants and resting-state fMRI data

Participants were selected from the Human Connectome Project (HCP) ‘PTN’ (Parcellation+Timeseries+Netmats) dataset (http://www.humanconnectome.org). These participants are healthy young adults within an age range of 22–37 years, which corresponds to a period after the completion of major neurodevelopment and before the onset of neurodegenerative changes. All 1003 subjects had complete resting-state fMRI data. Specifically, data from the earliest 184 subjects were reconstructed using an initial version of the data reconstruction software (referred to as ‘recon1’). Data from the latest 812 subjects were reconstructed using a later, slightly improved version of the data reconstruction software (referred to as ‘recon2’). Data from seven subjects were processed using a mixture of the two methods. To ensure data accuracy and consistency, we only used data from the latest 812 subjects that were reconstructed using the improved ‘recon2’ version. Each subject underwent four resting-state fMRI scans where subjects were instructed to keep their eyes open and move as little as possible (14.4 min/scan). The four fMRI scans were concatenated into continuous time series consisting of 4800 time points at a repetition time of 0.72 s. The full details regarding the sample and data acquisition have been reported in prior publications (Van Essen *et al.,* 2012, 2013). The HCP scanning protocol was approved by the Institutional Review Board of Washington University in St. Louis, MO, USA. Written informed consent was obtained from each participant.

### fMRI data preprocessing and construction of functional connectome

All resting-state fMRI data were minimally preprocessed with echo planar imaging gradient distortion correction, motion correction, field bias correction, spatial transformation and normalization into a common Montreal Neurological Institute space (Glasser *et al.,* 2013) and artifact removal using independent component analysis (ICA) + FIX (Salimi-Khorshidi *et al.,* 2014). For functional network connectivity analysis, network nodes can be defined by using existing atlases based on cytoarchitecture or anatomy. However, a potential pitfall in using such atlases is that the mean time series of a node may not represent any of the constituent time series if different functional areas are included within a single node (Shen *et al.,* 2013). Therefore, group-level ICA was used here to define the whole-brain network nodes in a data-driven fashion, which are considered more functional homogeneous and may be better at capturing individual differences of real functional boundaries than those defined by existing atlases (Calhoun *et al.,* 2001). The group-level ICA parcellation was performed using FSL’s MELODIC tool (Beckmann and Smith, 2004) and spatial-ICA was applied at several different dimensionalities (15, 25, 50, 100, 200 and 300). The dimensionality determines the number of ICA components; a higher number typically means that the significant areas within the spatial component maps will be smaller. Given that larger spatial components lack regional specificity, we used 100, 200 and 300 group-ICA components to define brain network nodes. That is, 200 components were used for the main analyses, and 100 and 300 components were used for the validation analyses. The locations of ICA-derived nodes were determined based on the peak coordinates in the ICA weight maps. For each node, one representative time series was derived by mapping the corresponding ICA spatial map onto each participant’s fMRI data using the standard ‘dual-regression stage-1’ approach, in which the ICA map was used as a spatial regressor against the full time series data. This resulted in 200 nodes’ time series that can be used to construct functional connectome at the individual level. Specifically, the partial temporal correlation coefficients between the time series of all possible pairs of nodes were computed, which estimate direct connection strengths better than achieved by Pearson’s correlation. The resultant correlation values were converted into *z* statistics with Fisher’s *r*-to-*z* transformation, resulting in a symmetric 200 × 200 connectivity matrix in which each element represents the strength of connection between two nodes (hereafter referred to as an edge).

### Personality assessment

Within the HCP behavioral measurements, the 60 item version of the Costa and McCrae Neuroticism/Extraversion/Openness Five Factor Inventory (NEO-FFI), which is a self-report questionnaire with excellent reliability and validity (McCrae and Costa, 2004), was administered to each participant to capture the major facets of human personality: neuroticism, extraversion, agreeableness, openness and conscientiousness. For each item, participants reported their agreement level on a five-point Likert scale, where the scores are derived by coding each item’s answer (strongly disagree = 0; disagree = 1; neither agree nor disagree = 2; agree = 3; strongly agree = 4) and then reverse coding appropriate items. We used the total score on each personality factor to search the edges containing information relevant for the subsequent prediction analyses. Only 810 subjects (408 female) were used in this study because 2 participants were excluded due to incomplete item-level personality data.

### Connectome-based predictive modeling

CPM is a recently developed approach for identifying brain networks associated with a behavioral variable of interest from whole-brain functional connectivity, which can be then used to predict novel participants’ behavior at the single-subject level (Shen *et al.,* 2017). Here, CPM was performed using previously validated custom MATLAB scripts, which are freely available online (https://www.nitrc.org/projects/bioimagesuite/). Overall, CPM took edge weights (i.e. whole-brain functional connectivity matrix) and behavioral data (i.e. the total score on each personality factor) as input to generate a predictive model of the behavior from edge. In the training set, behavior data were correlated with each edge using Pearson’s correlation analyses with a statistical significance threshold of *P* < 0.01 to identify positive and negative predictive networks. For positive networks, edge weights are significantly positively associated with the behavior; for negative networks, edge weights are significantly negatively associated with the behavior. Both networks are independent in predicting the same behavioral variable, because a single edge is either a positive or a negative predictor. Next, a single-subject summary value was created by summing the significant edge weights in each network and was then used to build a predictive model that assumes a linear relationship between the single-subject summary value of connectivity data (independent variable) and the behavioral variable (dependent variable). Finally, the resultant models were applied to the testing set to predict behavioral variables. Here, to take into account the family structure of the HCP cohort, we employed a leave-one-family-out cross-validation analysis (i.e. internal validation) to test whether the functional connectivity model could reliably predict personality factor scores in novel participants. Briefly, predicted scores of the participants within a left-out family were generated by the predictive model that was trained on the data from all other participants in an iterative manner until all participants had a predicted score. Model performance was assessed by the magnitude and statistical significance of the Pearson’s correlation between actual and predicted behavioral values. To account for the non-independence of analyses in the leave-one-family-out folds, we conducted nonparametric permutation testing instead of parametric testing to assess the statistical significance. To generate an empirical null distribution of the test statistic (i.e. prediction correlation values), we randomly shuffled the correspondence between connectivity matrices and behavioral variables 5000 times and reran the CPM pipeline using the shuffled data. Based on the null distribution, the *P* value for the leave-one-family-out prediction was calculated as the proportion of sampled permutations that were greater than or equal to the true prediction correlation, i.e. *P* value = the number of permutations that generated correlation values greater than or equal to the true correlation values/5000. Statistical significance was set at *P* < 0.05.

### Validation analyses

The following procedures were conducted to further evaluate the reproducibility of our findings. First, a significance threshold of *P* < 0.01 was used to select edges that were positively and negatively correlated with personality factors. To determine whether our main results depended on the choice of different thresholds, we reran the CPM analyses using two other thresholds (i.e. *P* < 0.05 and 0.001) to identify edges significantly related to personality factors. Second, considering that different parcellation strategies may influence the results, we constructed functional connectome using two other parcellation schemes (i.e. 100 and 300 group-ICA components) and repeated the entire analyses. Third, as several demographic (age and gender) and behavioral (intelligence) data and head motion could affect the functional connectivity–personality relationship, we performed the prediction analyses again with controlling for these confounding factors, i.e. personality factors were correlated with each edge using partial correlation analyses adjusting for age, gender, intelligence (PMAT24_A_CR) and overall head motion parameters. Fourth, despite evidence for the advantage of partial correlation over Pearson’s correlation in measuring functional connectivity, we also repeated the CPM analyses based on Pearson’s correlation functional connectivity to compare their prediction performances. Finally, we also performed personality prediction using a multivariate approach based on elastic-net algorithm (default hyperparameters: *alpha* = 1.0, *l1_ratio* = 0.5; leave-one-family-out cross-validation).

## Results

### Prediction performances of personality factors

**Fig. 1:**
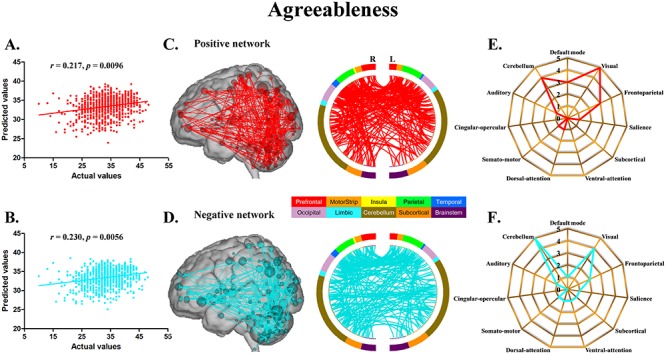
CPM of agreeableness. (A, B) Scatter plots showing the correspondence between actual (*x*-axis) and predicted (*y*-axis) agreeableness values generated using CPM based on the positive and negative networks. (C, D) High-degree nodes (degree ≥6, larger spheres indicate nodes with higher degree) and their connections in the positive and negative networks. (E, F) Polar plots illustrating the 20 highest degree nodes summarized by overlap with canonical neural networks in the positive and negative networks.

**Fig. 2:**
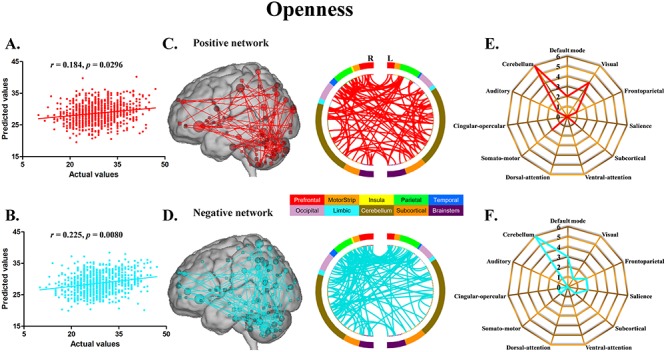
CPM of openness. (A, B) Scatter plots showing the correspondence between actual (*x*-axis) and predicted (*y*-axis) openness values generated using CPM based on the positive and negative networks. (C, D) High-degree nodes (degree ≥4, larger spheres indicate nodes with higher degree) and their connections in the positive and negative networks. (E, F) Polar plots illustrating the 20 highest degree nodes summarized by overlap with canonical neural networks in the positive and negative networks.

**Fig. 3:**
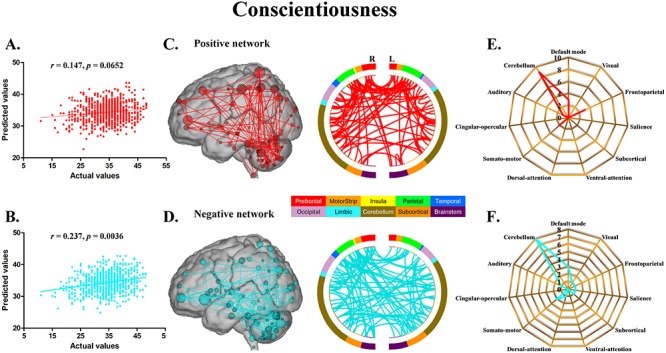
CPM of conscientiousness. (A, B) Scatter plots showing the correspondence between actual (*x*-axis) and predicted (*y*-axis) conscientiousness values generated using CPM based on the positive and negative networks. (C, D) High-degree nodes (degree ≥4, larger spheres indicate nodes with higher degree) and their connections in the positive and negative networks. (E, F) Polar plots illustrating the 20 highest degree nodes summarized by overlap with canonical neural networks in the positive and negative networks.

**Fig. 4:**
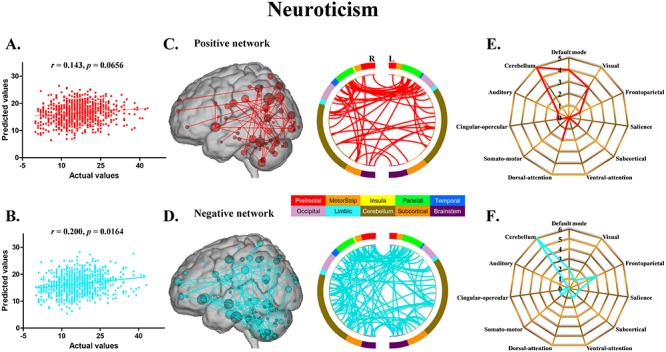
CPM of neuroticism. (A, B) Scatter plots showing the correspondence between actual (*x*-axis) and predicted (*y*-axis) neuroticism values generated using CPM based on the positive and negative networks. (C, D) High-degree nodes (degree ≥4, larger spheres indicate nodes with higher degree) and their connections in the positive and negative networks. (E, F) Polar plots illustrating the 20 highest degree nodes summarized by overlap with canonical neural networks in the positive and negative networks.

**Fig. 5:**
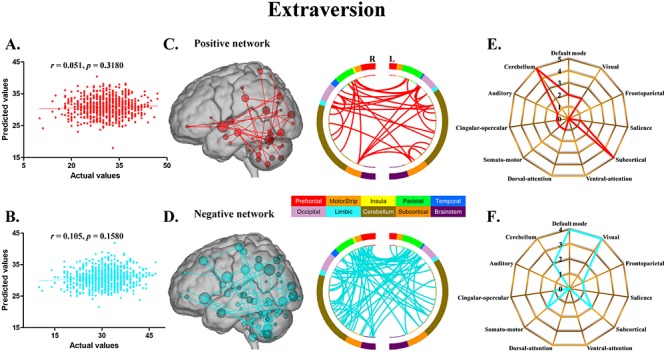
CPM of extraversion. (A, B) Scatter plots showing the correspondence between actual (*x*-axis) and predicted (*y*-axis) extraversion values generated using CPM based on the positive and negative networks. (C, D) High-degree nodes (degree ≥4, larger spheres indicate nodes with higher degree) and their connections in the positive and negative networks. (E, F) Polar plots illustrating the 20 highest degree nodes summarized by overlap with canonical neural networks in the positive and negative networks.

The CPM models, using functional connectivity within both the positive and negative networks, successfully predicted agreeableness (positive network: *r* = 0.217, *P* = 0.0096; negative network: *r* = 0.230, *P* = 0.0056) (Figure 1A and B) and openness (positive network: *r* = 0.184, *P* = 0.0296; negative network: *r* = 0.225, *P* = 0.0080) (Figure 2A and B). The CPM models based on the negative networks effectively predicted conscientiousness (negative network: *r* = 0.237, *P* = 0.0036) (Figure 3B) and neuroticism (negative network: *r* = 0.200, *P* = 0.0164) (Figure 4B), while the models based on the positive networks were marginally significant in predicting conscientiousness (positive network: *r* = 0.147, *P* = 0.0652) (Figure 3A) and neuroticism (positive network: *r* = 0.143, *P* = 0.0656) (Figure 4A). However, the CPM predictability of extraversion was low and did not reach statistical significance (positive network: *r* = 0.051, *P* = 0.3180; negative network: *r* = 0.105, *P* = 0.1580) (Figure 5A and B).

### Network anatomy

Because of the nature of cross-validation, it is likely that a slightly different set of edges will be selected as features in each iteration of the cross-validation. For illustrational purposes, we defined final personality factor-relevant networks using data from the entire sample, that is, personality factors were correlated with whole-brain functional connectivity in all 810 subjects to identify significant edges comprising positive and negative predictive networks. Overall, network anatomies for the networks associated with five personality factors were complex and included edges between nodes across the brain.

For agreeableness, the positive and negative networks consisted of 354 and 346 edges, respectively (Figure 1C and D). Highest degree nodes (i.e. nodes with the most edges) for the positive network included nodes belonging to default mode network (DMN), visual network (VN), frontoparietal network (FPN) and cerebellum; highest degree nodes for the negative network included nodes belonging to VN, FPN and cerebellum (Figure 1E and F). For openness, the positive and negative networks consisted of 175 and 178 edges, respectively (Figure 2C and D). Highest degree nodes for the positive network included nodes belonging to DMN, VN, somatomotor network (SMN) and cerebellum; highest degree nodes for the negative network included nodes belonging to DMN, FPN, salience network and cerebellum (Figure 2E and F). For conscientiousness, the positive and negative networks consisted of 172 and 187 edges, respectively (Figure 3C and D). Highest degree nodes for the positive network included nodes belonging to DMN, FPN, SMN and cerebellum; highest degree nodes for the negative network included nodes belonging to DMN, SMN and cerebellum (Figure 3E and F). For neuroticism, the positive and negative networks consisted of 135 and 176 edges, respectively (Figure 4C and D). Highest degree nodes for the positive network included nodes belonging to DMN, VN, dorsal attention network and cerebellum; highest degree nodes for the negative network included nodes belonging to DMN, FPN and cerebellum (Figure 4E and F). For extraversion, the positive and negative networks consisted of 141 and 148 edges, respectively (Figure 5C and D). Highest degree nodes for the positive network included nodes belonging to DMN, VN, subcortical network, and cerebellum; highest degree nodes for the negative network included nodes belonging to DMN, VN, SCN, SMN and cerebellum (Figure 5E and F).

### Validation analysis

First, using edges selected by thresholds of *P* < 0.05 and 0.001, we found that the prediction performances of personality factors were similar to those at the threshold of *P* < 0.01 but with a reduced degree (Supplementary Figures S1A–S5A and S1B–S5B). Second, we found that our main results were reproducible after considering the effects of different parcellation strategies, that is, agreeableness, openness, conscientiousness and neuroticism yielded higher predictability than extraversion across results derived from 100 and 300 group-ICA components (Supplementary Figures S1C–S5C and S1D–S5D). Third, the patterns of prediction results held although some findings lost significance after controlling for age, gender, intelligence and head motion (Supplementary Figures S1E–S5E). Fourth, based on Pearson’s correlation functional connectivity, the correlation coefficients between actual and predicted personality factors were lower than those based on partial correlation functional connectivity in the main analyses (Supplementary Figures S1F–S5F), suggesting the advantage of partial correlation over Pearson’s correlation in predicting personality. Finally, using a multivariate approach based on elastic-net algorithm yielded poorer prediction of agreeableness but better prediction of extraversion than using CPM, with prediction results of other personality factors (i.e. openness, conscientiousness and neuroticism) comparable to CPM (Fig. S6).

## Discussion

By applying a recently developed CPM approach to a large sample of high-quality resting-state fMRI data from the HCP, our study demonstrated that all the FFM personality factors (agreeableness, openness, conscientiousness and neuroticism) other than extraversion can be successfully and reliably predicted from an individual’s unique whole-brain functional connectivity profile. At the neural level, we found that the personality-associated functional networks mainly included brain regions within default mode, frontoparietal executive control, visual and cerebellar systems. These findings suggest that advances in neuroimaging techniques and analysis methods have made it increasingly feasible to translate brain imaging findings into real-world personality psychological settings.

Despite using the same CPM approach, prediction results of the FFM personality factors differ between the current report and the previous studies (Dubois *et al.,* 2018; Hsu *et al.,* 2018). The discrepancies between our study and Hsu et al.’s study might arise from sample selection (e.g. 810 healthy young subjects with an age range of 22–37 years *vs* 114 subjects with an age range of 18–85 years), differences in fMRI data acquisition [e.g. repetition time (TR) = 720 ms *vs* TR = 1400 and 645 ms] and preprocessing (e.g. artifact removal using ICA + FIX *vs* global signal regression), different whole-brain parcellation schemes [i.e. 100, 200 and 300 nodes based on group-level ICA *vs* 268 nodes based on the predefined Shen *et al.*’s (2013) brain atlas] and difference in functional connectivity calculation (partial correlation *vs* Pearson’s correlation). The disparities between our study and Dubois et al.’s study may be explained by difference in fMRI data preprocessing (i.e. the HCP minimal preprocessing pipeline *vs* the own preprocessing procedure), different whole-brain parcellation strategies [i.e. data-driven parcellation using group-level ICA *vs* hard parcellation using the existing Shen *et al.*’s (2013) brain atlas] and difference in functional connectivity calculation (partial correlation *vs* Pearson’s correlation). Notably, we attribute these inconsistent findings mainly to the differences in brain parcellation and functional connectivity calculation. Although there is no consensus on the best parcellation for whole-brain functional connectivity analysis, the ICA-derived more homogeneous and functionally coherent regions may contribute to a significant prediction of personality factors observed in the present study. However, we did not conduct a validation analysis with hard parcellation because of the difficulties in downloading and processing the huge resting-state fMRI data from HCP. With respect to functional connectivity calculation, we found that partial correlation used in the main analyses resulted in a better personality prediction than Pearson’s correlation in the validation analyses.

DMN primarily consists of medial prefrontal cortex (MPFC), posterior cingulate cortex/precuneus (PCC/PCu), inferior parietal lobule (IPL) and lateral temporal cortex (LTC). DMN is preferentially active when individuals are engaged in spontaneous and self-generated cognition (Buckner *et al.,* 2008; Raichle, 2015). Previous neuroimaging studies have provided evidence that structure and function of DMN are closely linked to personality. For example, a large sample study based on surface-based morphometry (SBM) revealed that higher openness was associated with greater surface area in IPL and greater cortical folding in PCC; higher conscientiousness with greater cortical thickness in PCu, smaller surface area in LTC and smaller cortical folding in LTC and MPFC; higher neuroticism with smaller surface area in LTC and smaller cortical folding in MPFC and LTC; higher extraversion with greater cortical thickness in PCu and smaller surface area in LTC (Riccelli *et al.,* 2017b). Other moderate and small sample SBM studies have identified an association between higher neuroticism and smaller surface area in MPFC and LTC (Bjornebekk *et al.,* 2013), as well as a link between higher openness and lower cortical thickness in IPL (Wright *et al.,* 2007). Previous voxel-based morphometry (VBM) studies have found that smaller volume in MPFC is associated with lower extraversion (DeYoung *et al.,* 2010; Kapogiannis *et al.,* 2013) and higher neuroticism and openness (Kapogiannis *et al.,* 2013), smaller volume in LTC with lower extraversion (Kapogiannis *et al.,* 2013) and higher agreeableness (DeYoung *et al.,* 2010; Kapogiannis *et al.,* 2013), smaller volume in PCC with lower (DeYoung *et al.,* 2010) and higher (Coutinho *et al.,* 2013) agreeableness and smaller volume in IPL with higher agreeableness (Coutinho *et al.,* 2013). In a study of teenagers, higher neuroticism was found to correlate with greater MPFC volume and cortical thickness in females, while the correlations exhibited an opposite effect in males (Blankstein *et al.,* 2009). Another VBM study on the role of gender demonstrated that greater volume in PCu was associated with higher extraversion and conscientiousness in males rather than in females (Nostro *et al.,* 2017). A longitudinal study showed that subjects with a personality trait of less openness had an accelerated loss of gray matter volume in IPL (Taki *et al.,* 2013). Ferschmann et al. found that higher conscientiousness was associated with slower annual percentage change of cortical thickness in MPFC and PCu across adolescence (Ferschmann *et al.,* 2018). With regard to DMN function, a prior task fMRI study reported a correlation between higher conscientiousness and increased MPFC activation in response to an oddball task (Eisenberger *et al.,* 2005). A resting-state fMRI study of the relationship between DMN and personality demonstrated that different personality factors were associated with activity in different DMN components, i.e. extraversion and agreeableness related to the midline core of DMN, while neuroticism, openness and conscientiousness related to the parietal cortex system (Sampaio *et al.,* 2014). Gentili *et al.* (2017) observed that neuroticism was correlated with several resting-state functional measures in multiple regions of DMN. Regional neural activity analyses have revealed that lower extraversion is associated with lower activity in MPFC (Wei *et al.,* 2014) and PCu (Kunisato *et al.,* 2011; Wei *et al.,* 2014) and lower neuroticism with higher activity in PCu (Kunisato *et al.,* 2011). Previous seed-based functional connectivity studies have identified links between personality traits and functional connectivity of DMN seeds (e.g. PCC and PCu) (Adelstein *et al.,* 2011; Ryan *et al.,* 2011). In addition, Aghajani et al. reported that higher neuroticism was correlated with increased functional connectivity between amygdalar seed and DMN hubs (Aghajani *et al.,* 2014). Moreover, a graph theoretical study of functional brain network indicated that higher openness was associated with higher global efficiency of DMN (Beaty *et al.,* 2016).

FPN, which is involved in a variety of cognitive-control processes (Cole and Schneider, 2007; Xin and Lei, 2015), primarily consists of dorsolateral and dorsomedial prefrontal cortex (DLPFC and DMPFC), posterior parietal cortex (PPC) and frontal eye fields (FEF). There is a large body of evidence in support of the association between FPN and personality. In the large-scale cohort study by Riccelli et al., higher agreeableness was found to be associated with smaller cortical thickness in DLPFC; higher openness with smaller cortical thickness in DLPFC; higher conscientiousness with greater cortical thickness in DLPFC; higher neuroticism with greater cortical thickness in DLPFC and PPC, smaller surface area in DLPFC and smaller cortical folding in DLPFC and PPC (Riccelli *et al.,* 2017b). Although many structural MRI studies of small-to-moderate samples have yielded mixed findings, DLPFC morphology measured by volume (DeYoung *et al.,* 2010; Coutinho *et al.,* 2013; Kapogiannis *et al.,* 2013; Lu *et al.,* 2014), cortical thickness (Wright *et al.,* 2007), surface area (Bjornebekk *et al.,* 2013) and cortical folding (Schultz *et al.,* 2017) has been consistently shown to relate to multiple personality dimensions. A longitudinal study of brain development reported that higher conscientiousness was associated with slower annual percentage change of cortical thickness in DLPFC and PPC across adolescence (Ferschmann *et al.,* 2018). Using task fMRI, investigators have demonstrated that personality traits are correlated with neural activation or functional/effective connectivity in FPN during a broad range of tasks involving negative emotional facial expressions (Cremers *et al.,* 2010), oddball (Eisenberger *et al.,* 2005) and working memory (Dima *et al.,* 2015). In addition, a resting-state fMRI study reported that higher regional activity of DLPFC was associated with lower neuroticism and openness and higher extraversion and conscientiousness (Kunisato *et al.,* 2011). Moreover, individuals with focal damage to DLPFC were found to exhibit personality changes including higher neuroticism and lower conscientiousness (Forbes *et al.,* 2014).

VN, known to be implicated in visual perception and processing (Grill-Spector and Malach, 2004; Golarai *et al.,* 2007), is centered on medial occipital cortex (lingual gyrus, cuneus and calcarine sulcus), lateral occipital cortex (LOC) and fusiform gyrus (FFG). Researchers have found that VN plays a pivotal role in some personality domains. By using SBM, Riccelli et al. observed that higher agreeableness was associated with smaller surface area in FFG; higher openness with greater surface area in LOC and greater cortical folding in cuneus; higher conscientiousness with smaller surface area in LOC and smaller cortical folding in LOC and FFG; higher neuroticism with smaller surface area in cuneus and smaller cortical folding in LOC; higher extraversion with greater cortical folding in FFG (Riccelli *et al.,* 2017b). VBM studies have revealed that smaller volume in FG was linked to higher conscientiousness and lower agreeableness (DeYoung *et al.,* 2010) and lower volume in LOC to higher agreeableness (Coutinho *et al.,* 2013). A prior study investigating the effect of gender on personality–brain structure relationship showed that lower volume in cuneus and FG was associated with higher neuroticism and lower extraversion in males rather than in females (Nostro *et al.,* 2017). In the longitudinal study by Ferschmann *et al.* (2018), higher conscientiousness was found to correlate with slower annual percentage change of surface area in lingual gyrus across adolescence. As to brain function, Gentili *et al.* (2017) reported that neuroticism was associated with several resting-state functional metrics in multiple VN regions. Furthermore, seed-based resting-state functional connectivity studies have consistently identified links between amygdala-VN connectivity and personality traits, such as extraversion (Aghajani *et al.,* 2014) and neuroticism (Kruschwitz *et al.,* 2015).

It has now become apparent that cerebellum is engaged in multiple high-order functions (Schmahmann and Sherman, 1998; Stoodley and Schmahmann, 2010) and its role in understanding human personality has also been evident. For instance, previous structural MRI studies have yielded a consistent finding that higher neuroticism is linked to greater volume in cerebellum (DeYoung *et al.,* 2010; Lu *et al.,* 2014). Using VBM, Nostro *et al.* (2017) found that lower volume in cerebellum was associated with higher neuroticism and lower extraversion in males, while the correlations were absent in females, which underlines the important role of gender in personality–brain structure associations. Additionally, resting-state fMRI research has shown that higher regional activity of cerebellum correlates with lower conscientiousness (Kunisato *et al.,* 2011). Moreover, individuals with cerebellar lesions have also been found to show personality alterations (Marien *et al.,* 2009; Stoodley and Schmahmann, 2010).

Our study has several limiting factors that should be mentioned. First, our data do not allow inference on causality between personality factors and brain functional connectivity, which likely involves complex interactions of different neuropsychological mechanisms that remain to be fully elucidated. Second, the lack of data from an independent sample precludes us from conducting an external validation analysis. Third, we did not identify reliable prediction of extraversion, and the reason for the null findings still needs to be further explored. By one view, extraversion is a complex personality trait that might be predicted from higher order functional network measures (e.g. topological properties from graph theory), rather than simple functional connectivity. An alternate possibility is that there is a complex relationship between extraversion and functional connectivity beyond a simple linear correlation. Thus, nonlinear models may be more appropriate than CPM linear models. However, the difficulty in using nonlinear models is that a much larger number of training samples than the number of features are required. Collectively, this issue should be more fully addressed in future studies. Finally, the prediction of some personality factors (agreeableness and neuroticism) attenuated to nominal significance after correction for multiple comparisons. However, because our prediction analyses are exploratory and the preliminary results may contribute to a better understanding of the nature and extent of the associations between personality and functional connectivity, the prediction results without correction for multiple comparisons were reported in this study.

In conclusion, our large sample study demonstrates that resting-state functional connectivity patterns of whole-brain large-scale networks can effectively and reliably predict complex human personality traits, including agreeableness, openness, conscientiousness and neuroticism, at the individual level. Our data also demonstrate that individual differences in connectivity of default mode, executive control, visual and cerebellar systems contribute most to variability in personality. These findings may help advance the translation of ‘brain connectivity fingerprinting’ into real-world settings of personality or other complex social, cognitive or affective constructs.

## Conflict of interest

There is no conflict of interest to declare.

## Funding

The work was supported by the National Natural Science Foundation of China (grant numbers: 81801679, 81571308 and 81771817). Data were provided by the Human Connectome Project, WU-Minn Consortium (Principal Investigators: David Van Essen and Kamil Ugurbil; 1U54MH091657) funded by the 16 NIH Institutes and Centers that support the NIH Blueprint for Neuroscience Research and by the McDonnell Center for Systems Neuroscience at Washington University.

## Supplementary Material

scan-19-347-File007_nsaa044
