# Supplementary material for: Robust prediction of individual personality from brain functional connectome
Source: Soc Cogn Affect Neurosci. 2020 Apr 4;15(3):359–69. doi: 10.1093/scan/nsaa044 (PMC7235956; doi:10.1093/scan/nsaa044)
Supplement: scan-19-347-File007_nsaa044 [file scan-19-347-file007_nsaa044.doc]

**Supplementary Materials**


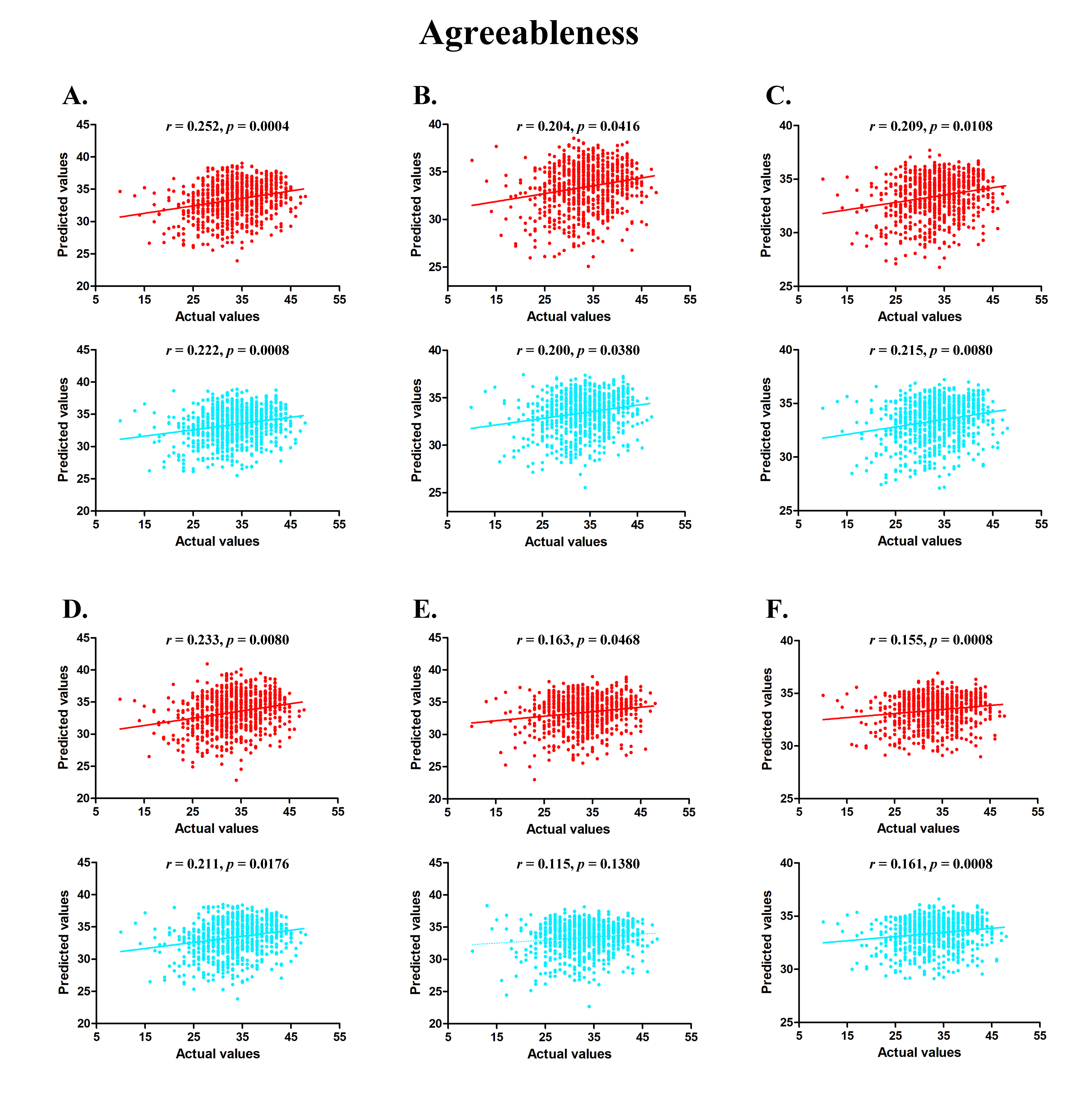


**Figure S1.** Scatter plots showing the correspondence between actual (x-axis) and predicted (y-axis) agreeableness generated from CPM using a leave-one-family-out cross-validation scheme. (A) Edges selection threshold of *p* < 0.05. (B) Edges selection threshold of *p* < 0.001. (C) 100 group-ICA components. (D) 300 group-ICA components. (E) Controlling for age, gender, intelligence, and head motion. (F) Pearson’s correlation functional connectivity. Abbreviations: CPM, connectome-based predictive modeling; ICA, independent component analysis.


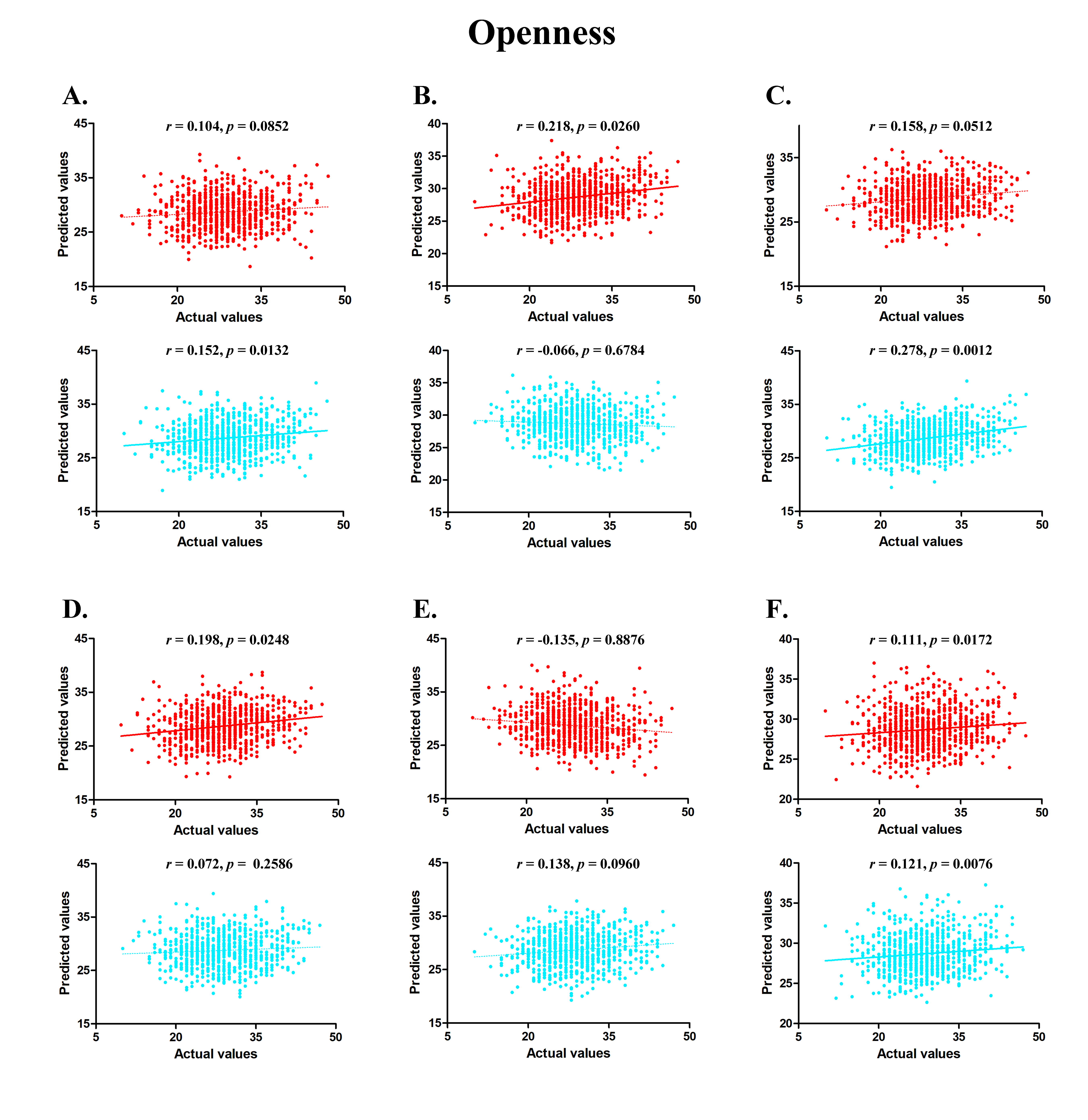


**Figure S2.** Scatter plots showing the correspondence between actual (x-axis) and predicted (y-axis) openness generated from CPM using a leave-one-family-out cross-validation scheme. (A) Edges selection threshold of *p* < 0.05. (B) Edges selection threshold of *p* < 0.001. (C) 100 group-ICA components. (D) 300 group-ICA components. (E) Controlling for age, gender, intelligence, and head motion. (F) Pearson’s correlation functional connectivity. Abbreviations: CPM, connectome-based predictive modeling; ICA, independent component analysis.


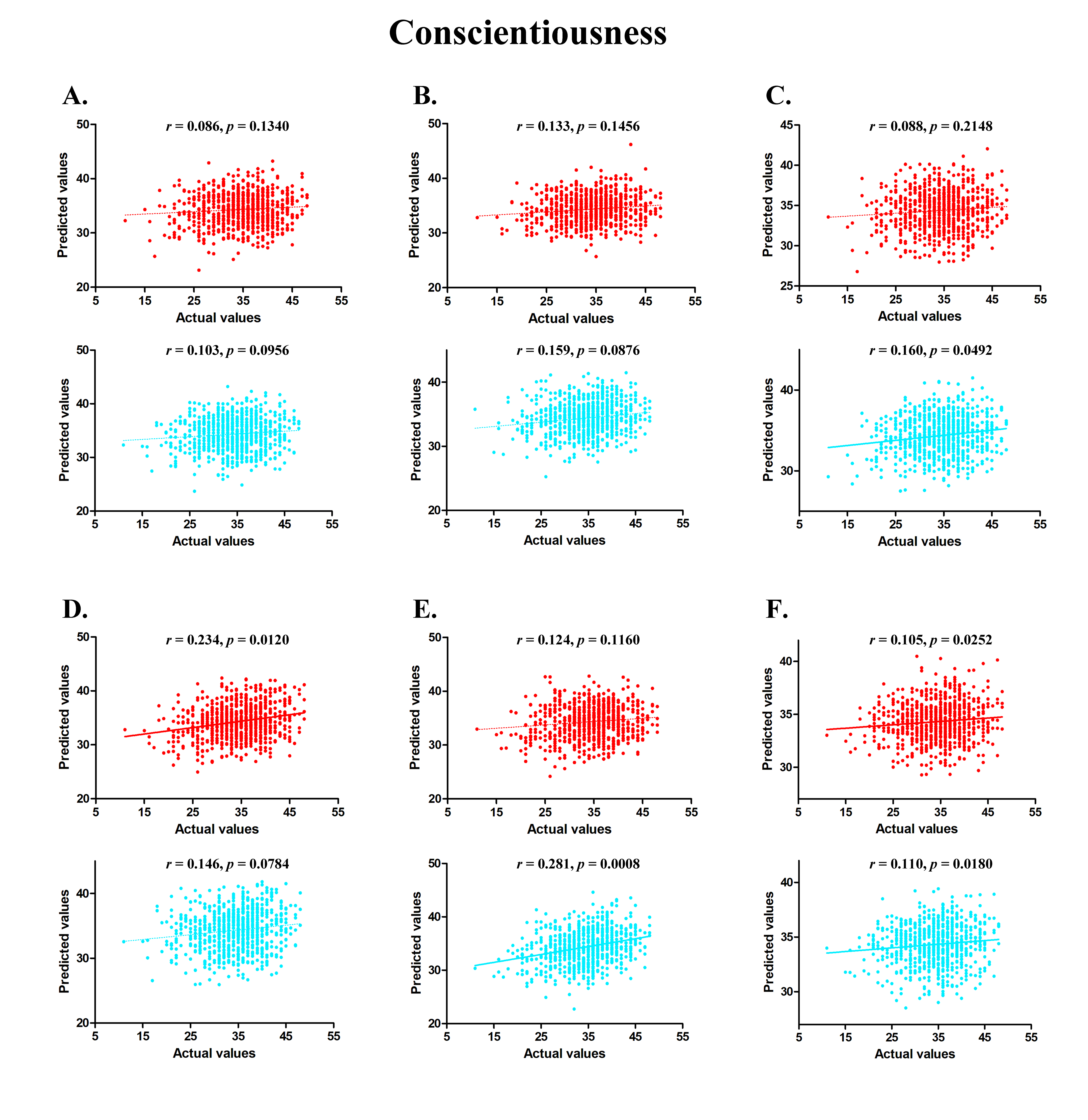


**Figure S3.** Scatter plots showing the correspondence between actual (x-axis) and predicted (y-axis) conscientiousness generated from CPM using a leave-one-family-out cross-validation scheme. (A) Edges selection threshold of *p* < 0.05. (B) Edges selection threshold of *p* < 0.001. (C) 100 group-ICA components. (D) 300 group-ICA components. (E) Controlling for age, gender, intelligence, and head motion. (F) Pearson’s correlation functional connectivity. Abbreviations: CPM, connectome-based predictive modeling; ICA, independent component analysis.


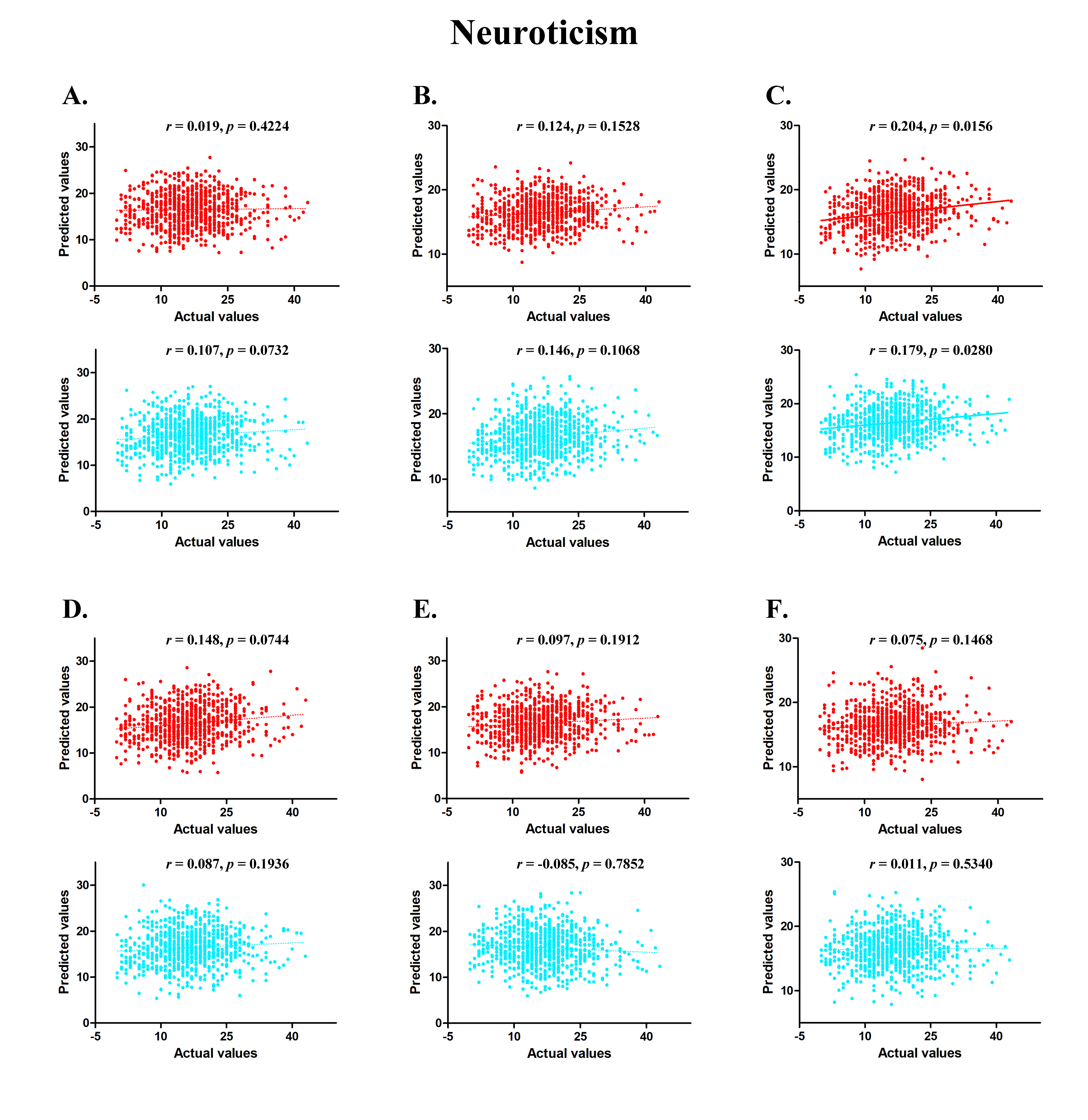


**Figure S4.** Scatter plots showing the correspondence between actual (x-axis) and predicted (y-axis) neuroticism generated from CPM using a leave-one-family-out cross-validation scheme. (A) Edges selection threshold of *p* < 0.05. (B) Edges selection threshold of *p* < 0.001. (C) 100 group-ICA components. (D) 300 group-ICA components. (E) Controlling for age, gender, intelligence, and head motion. (F) Pearson’s correlation functional connectivity. Abbreviations: CPM, connectome-based predictive modeling; ICA, independent component analysis.


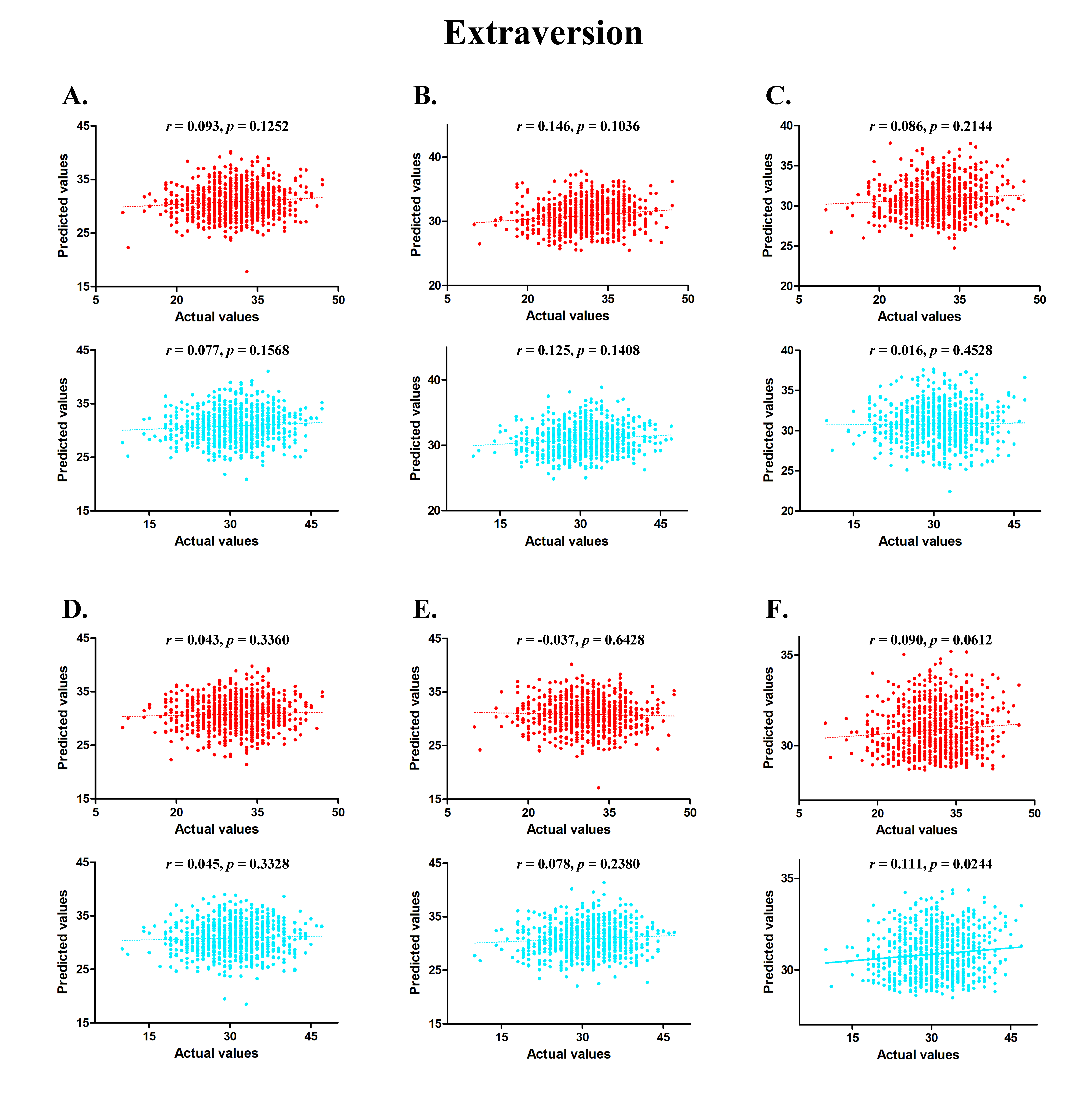


**Figure S5.** Scatter plots showing the correspondence between actual (x-axis) and predicted (y-axis) extraversion generated from CPM using a leave-one-family-out cross-validation scheme. (A) Edges selection threshold of *p* < 0.05. (B) Edges selection threshold of *p* < 0.001. (C) 100 group-ICA components. (D) 300 group-ICA components. (E) Controlling for age, gender, intelligence, and head motion. (F) Pearson’s correlation functional connectivity. Abbreviations: CPM, connectome-based predictive modeling; ICA, independent component analysis.


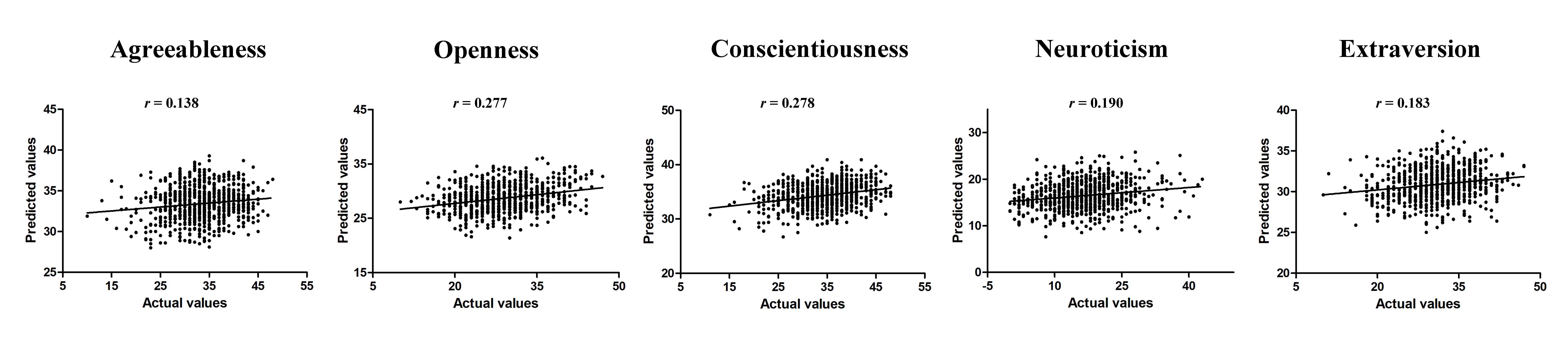


**Figure S6.** Scatter plots showing the correspondence between actual (x-axis) and predicted (y-axis) personality factors using a multivariate approach based on elastic-net algorithm.
